# Lung ultrasound may support internal medicine physicians in predicting the diagnosis, bacterial etiology and favorable outcome of community-acquired pneumonia

**DOI:** 10.1038/s41598-021-96380-x

**Published:** 2021-08-23

**Authors:** Filippo Mearelli, Chiara Casarsa, Alessandro Trapani, Pierlanfranco D’agaro, Cristina Moras, Francesca Spagnol, Federica Pellicori, Alessio Nunnari, Alice Massolin, Giulia Barbati, Gianni Biolo

**Affiliations:** 1 Unit of Clinica Medica, University of Trieste, Strada di Fiume 447, 34149 Trieste, Italy; 2 Department of Virology, University of Trieste, Trieste, Italy; 3 Biostatistics Unit, University of Trieste, Trieste, Italy

**Keywords:** Medical research, Epidemiology

## Abstract

To assess the usefulness of lung ultrasound (LUS) for identifying community-acquired pneumonia (CAP) among adult patients with suspected lower respiratory tract infection (LRTI) and for discriminating between CAP with different cultural statuses, etiologies, and outcomes. LUS was performed at internal medicine ward admission. The performance of chest X-ray (CXR) and LUS in diagnosing CAP in 410 patients with suspected LRTI was determined. All possible positive results for pneumonia on LUS were condensed into pattern 1 (consolidation + / − alveolar-interstitial syndrome) and pattern 2 (alveolar-interstitial syndrome). The performance of LUS in predicting culture-positive status, bacterial etiology, and adverse outcomes of CAP was assessed in 315 patients. The area under the receiver operating characteristic curve for diagnosing CAP by LUS was significantly higher than for diagnosis CAP by CXR (0.93 and 0.71, respectively; *p* < 0.001). Pattern 1 predicted CAP with bacterial and mixed bacterial and viral etiologies with positive predictive values of 99% (95% CI, 94–100%) and 97% (95% CI, 81–99%), respectively. Pattern 2 ruled out mortality with a negative predictive value of 95% (95% CI, 86–98%), respectively. In this study, LUS was useful in predicting a diagnosis of CAP, the bacterial etiology of CAP, and favorable outcome in patients with CAP.

## Introduction

Lower respiratory tract infection (LRTI) acquired in the community is one of the most common reasons for hospitalization among adults^1^. According to guidelines, the demonstration of an infiltrate on chest X-ray (CXR) in patients with LRTI acquired in the community suggests a diagnosis of community-acquired pneumonia (CAP)^2^. However, several studies point to CXR’s low sensitivity for diagnosing CAP^3–6^. There are increasing data on the use of lung ultrasound (LUS) to diagnose pneumonia^3–6^. Most of those works enrolled patients with a suspect of CAP which was neither standardised nor reproducible. This issue may impact massively both in the diagnostic performance of LUS and in the generalizability of the results. Additionally, even though ruling in/out pneumonia in patients with suspected LRTI acquired in community is a common issue in clinical practice, the diagnostic performances of CXR and LUS have never been compared in this cohort of patients^3–9^.

In many studies of adult patients with CAP, bacteria were shown to be the most commonly detected organisms^10–16^, while viruses were identified in approximately one-third of cases^17,18^. More recently, the recognition of viral pathogens in the etiology of CAP has increased^1^. At hospital admission, clinical features of bacterial or viral CAP frequently overlap and cannot be used reliably to distinguish between the two etiologies^19^. Even CXR findings are not sufficiently specific to determine the causative agents of infection and influence treatment decision. Finally, since CAP is associated with high mortality and morbidity, there is an urgent need for early detection of a severe disease course^13^.

If LUS was confirmed to be an accurate diagnostic method for identifying CAP among undifferentiated patients with suspected LRTIs, one might hypothesize that it could also be useful in helping physicians predict the etiology of CAP and CAP outcomes. However, studies do not exist that attempted to associate unique LUS features with these factors in patients in whom the etiology of CAP was established with reasonable certainty.

Therefore, we conducted a prospective study to answer to three questions:

- first, is LUS more useful than CXR to predict diagnosis of pneumonia among patients with a standardised and reproducible suspect of community-acquired LRTI ?
- second, is LUS helpful to predict the etiology of CAP in patients in whom the causative agents of pneumonia are established according to a broad microbiological work-up ?
- third, is LUS useful in predicting favorable/unfavorable outcome of CAP ?

## Materials and methods

Hospitalized adults aged ≥ 18 years with suspected LRTI^2^ were recruited in a 45-bed internal medicine ward (IMW) at the University Hospital of Trieste, Italy. Enrollment of the patients started on January 1, 2018, and ended on January 1, 2020 (see Sample Size paragraph of the Supplementary info). All patients underwent a clinical work-up at the discretion of the physician in charge. At IMW admission, LUS was performed by two internal medicine physicians (FM/CC) with a multiprobe machine (MyLab25 Gold—Esaote, Genoa, Italy). Both the anterior and posterior chest were studied with a 4- to 8-MHz linear probe and a 2.5- to 3.5-MHz curved array probe: the scanning technique were detailed in the Supplementary info. FM and CC have at least 8 years of experience with point-of-care ultrasonography; they were blinded to all clinical data (including the CXR results) except for the fact that LRTI was suspected. Before the start of enrollment, we decided to condense all possible positive LUS features for CAP into pattern 1 (one or more subpleural consolidations with or without one or more areas of alveolar-interstitial syndrome) and pattern 2 (one or more areas of alveolar-interstitial syndrome). Consolidation was adjudicated at LUS when the pleural line was no longer distinguishable but appeared interrupted and replaced by a tissue-like structure that was mobile with respiratory movements. This image could contain air bronchograms (multiple hyperechogenic lentil-sized spots) or fluid bronchograms (anechoic). Alveolar-interstitial syndrome was defined as the presence of three or more B-lines in a longitudinal plane between two ribs in a given lung region. LUS was considered positive for CAP when pattern 1 or pattern 2 was detected in any area of the two hemithoraxes. LUS was considered as negative for CAP when none of the two patterns were identified in any area of the two hemithoraxes. The radiologist in charge who performed CXR (and computed chest tomography [CCT]) was blinded to LUS results and all clinical data except for the fact that LRTI was suspected. CXR and CCT were considered positive when at least one typical consolidation was visualized^3–6^. At discharge or death, the definitive diagnosis of the acute process, extension and results of the clinical work-up, and eventual worsening of clinical conditions within 30 days of IMW admission (details available in the Supplementary info) in patients with a final diagnosis of CAP were reviewed. CAP patients were grouped in different cohorts according to the availability and results of a predefined minimum microbiological work-up (available in the Supplementary info, as are the definitions used to identify the different microbiological CAP cohorts). To facilitate the recognition of the main microbiological groups of CAP in the tables and figures, a specific color was assigned to each cohort. All methods were performed in accordance with the relevant guidelines and regulations.

### Objectives

First analysis: to determine whether CXR or LUS is the better radiological examination to diagnose CAP in patients with suspected LRTI.

Second analysis: to identify the LUS features that, at hospital admission, could be predictive of 1) a specific cultural status, 2) the need for empirical antibacterial therapy, and 3) the need to escalate/de-escalate the intensity of treatment in patients with a definitive diagnosis of CAP. The inclusion and exclusion criteria of the first and second analyses are available in the Supplementary info.

### Statistical analysis

Continuous data are expressed as medians (interquartile ranges) according to their distribution. Categorical variables are expressed as numbers and percentages. Pearson’s chi-square test was used to analyze categorical variables, and the Mann–Whitney test was used to analyze continuous variables. Bonferroni correction was applied when more than two groups were compared. In the first analysis, the number of positive and negative results for CAP of both CXR and LUS were put side by side in patients with suspected LRTI. The “gold standard” for CAP was the definitive diagnosis of CAP recorded by the physician in charge at the end of the clinical work-up^3–6^. The results of LUS were not incorporated into the final diagnosis assessment. For the second analysis, the frequency of each LUS result (positive/negative) and positive pattern (pattern 1 and pattern 2) were compared in the following cohorts: 1) patients with culture-positive CAP vs those with all culture-negative CAP, 2) patients with all bacterial CAP vs those with viral CAP and CAP due to coinfection vs those with viral CAP, and 3) deteriorating patients vs nondeteriorating patients with CAP and nonsurviving vs surviving patients with CAP. Since bacteria and pattern 1 were the most common pathogen and LUS pattern, respectively, in patients with CAP in this study (Table 1), we theorized that pattern 1 could be predictive of culture-positive CAP, all bacterial CAP, and CAP due to coinfection. The diagnostic performance of LUS and CXR in detecting CAP and the performance of pattern 1 in predicting all the outcomes of the second analysis were quantified using C statistics (the area under the receiver operating characteristic curve [AUROC], sensitivity, specificity, positive predictive values [PPVs], negative predictive values [NPVs], positive likelihood ratio, negative likelihood ratio, and respective 95% confidence intervals [CIs] were estimated). All the analyses were performed using SPSS statistical package, version 20.0 (Armonk, NY: IBM Corp).

**Table 1:** Patients with definitive diagnosis of community-acquired pneumonia: characteristics at internal medicine ward admission, lung ultrasound results and positive patterns, extension of the minimum microbiological work up, main microbiological cohorts, and outcome at 30 days.

Characteristics	n = 315 (100)
**Demographics**	
Male	167 (53)
Median age (IQR)	82 (76–88)
**Comorbidities**	
Diabetes	74 (23)
Chronic heart failure	87 (28)
Previous acute myocardial infarction	51 (16)
Cancer	
Solid	40 (13)
Haematologic	20 (6)
Chronic liver disease	3 (1)
Chronic pulmonary disease	125 (40)
Chronic kidney disease	61 (19)
Dementia	61 (19)
Chronic rheumatologic disease	8 (2)
**Laboratory results at IMW admission**	
Median procalcitonin (ng/ml) (IQR)	0.28 (0.08–1.44)
**LUS results and patterns**	
Positive LUS result	307 (97)
Pattern 1	249 (79)
Pattern 2	58 (19)
Negative LUS result	8 (3)
**Severity at IMW admission**	
Median number of organ dysfunction	2 (1–2)
**Minimum microbiological work up**	
At least two sets of blood cultures	315 (100)
At least one:	
Respiratory sample for cultures	196 (62)
Urine sample for pneumococcal and *Legionella* antigens	280 (89)
Serum sample for atypical bacteria and respiratory viruses (serologies)	260 (82)
Nasopharyngeal swab for respiratory viruses (PCR)	205 (65)
**Cultural status of CAP**	
Culture-positive CAP	93 (30)
All culture-negative CAP	133 (42)
CAP with unknown status of respiratory cultures	89 (28)
**Etiology of microbiologically documented CAP**	
Bacterial infection alone	
Bacterial CAP	90 (29)
Viral infection	
CAP due to coinfection	29 (48)
Viral CAP	19 (32)
Presumptive viral CAP	12 (20)
All bacterial CAP	119 (79)
**Outcome within 30 days of IMW admission**	
Worsening of clinical conditions	77 (24)
Mortality	53 (17)

*IQR* interquartile range, *IMW* internal medicine ward, *LUS* lung ultrasound, *PCR* polymerase chain reaction, *CAP* community-acquired pneumonia.

Please see Supplement for discursive definitions of each microbiological cohort of community-acquired pneumonia patients.

### Ethical approval and consent to participate

The study protocol was approved by the medical ethical committee of Trieste. Informed consent before study enrolment was obtained from all patients or their legal representatives or surrogates.

## Results

**Figure 1:**
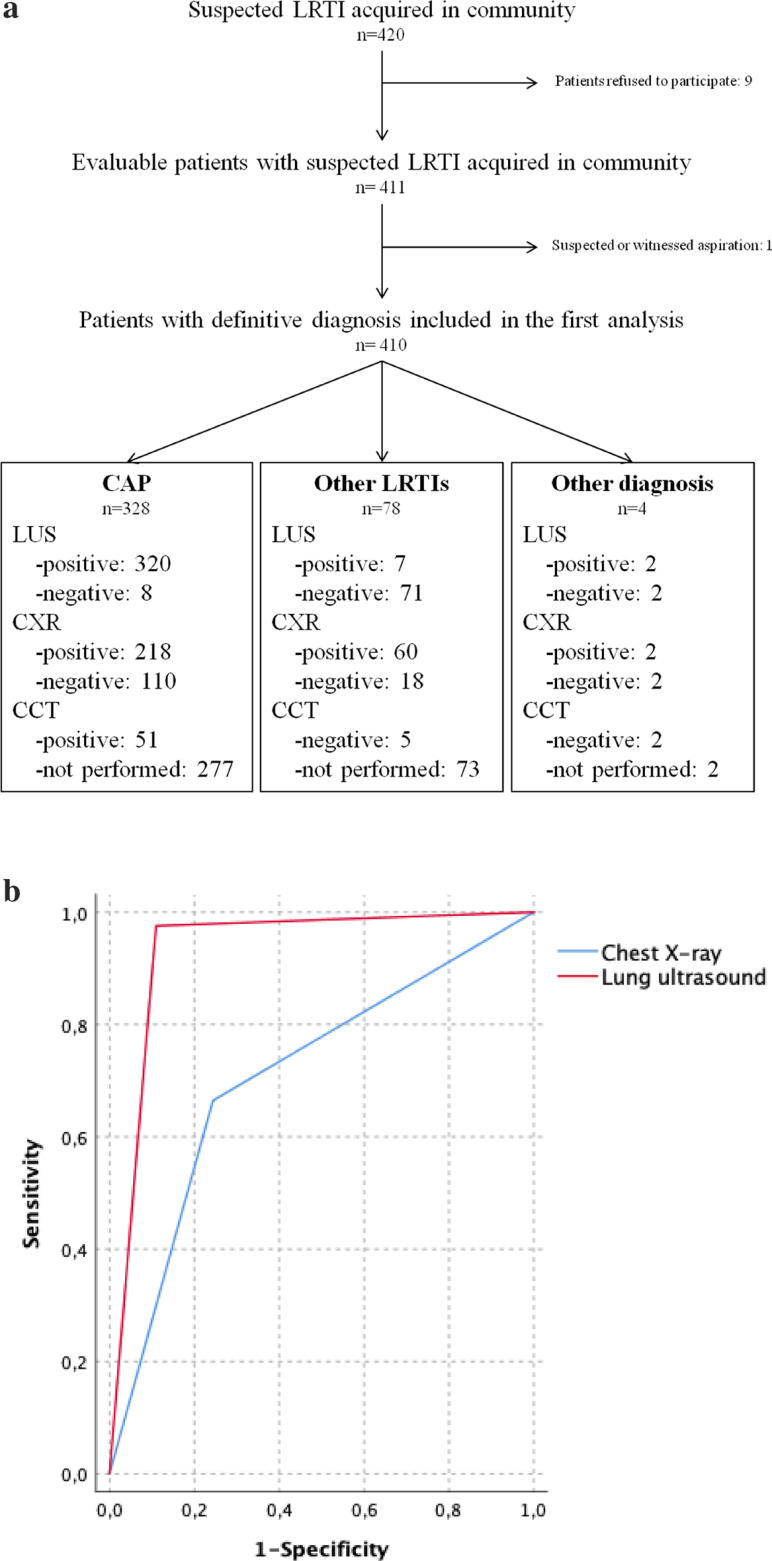
(**a**) Flow chart of participant enrollment and results of chest X-ray and lung ultrasound according to the definitive diagnosis made by physician in charge at the end of clinical work-up. List of abbreviations: *LRTI* lower respiratory tract infection, *CAP* community-acquired pneumonia, *LUS* lung ultrasound, *CXR* chest X-ray, *CCT* computed chest tomography. (**b**) Receiver operating characteristic curves for diagnosing pneumonia among patients with suspected lower respiratory tract infection by lung ultrasound and chest X-ray. Areas under the receiver operating characteristic curves = lung ultrasound: 0.93 (95% CI, 0.89–0.97), chest X-ray: 0.71 (95% CI, 0.65–0.77).

Of the 420 patients with suspected LRTI eligible for inclusion, 9 patients refused to participate. In 1 patient, aspiration was witnessed. A total of 410 patients underwent both CXR and LUS at IMW admission and were included in the first analysis (Fig. 1a); their clinical characteristics are shown in Table S1 (available in the Supplement). At the end of the clinical work-up, 328 (80%) patients received a definitive diagnosis of CAP. Other types of LRTI were diagnosed in 78 (19%) patients. The acute process was not proven to be LRTI in 4 (1%) patients. The positive and negative results of CXR and LUS according to the definitive diagnosis are reported in Fig. 1a. False positive and false negative LUS results were obtained in 9 and 8 patients, respectively. CCT was available in all the former and in none of the latter patients. The AUROC for diagnosing CAP by LUS was significantly higher than that for diagnosing CAP by CXR (0.93 and 0.71, respectively; *p* < 0.001. Figure 1b). The performance of CXR and LUS for ruling in/out CAP among patients with suspected LRTI is presented in Table 2.

**Table 2:**
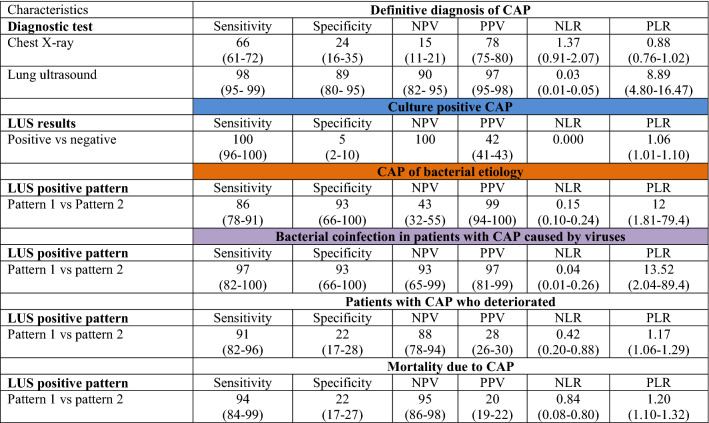
Performance of chest X-ray and lung ultrasound for predicting the definitive diagnosis of community-acquired pneumonia (first analysis) and performance of lung ultrasound for predicting outcomes in the second analysis.

*CXR* Chest X-Ray, *LUS* lung ultrasound, *CAP* community-acquired pneumonia, *NPV* negative predictive value, *PPV* positive predictive value, *NLR* negative likelihood ratio, *PLR* positive likelihood ratio.

The color of the heading refers to the color assigned to the specific microbiological cohort of community-acquired pneumonia patients in Figure S1.

**Figure 2:**
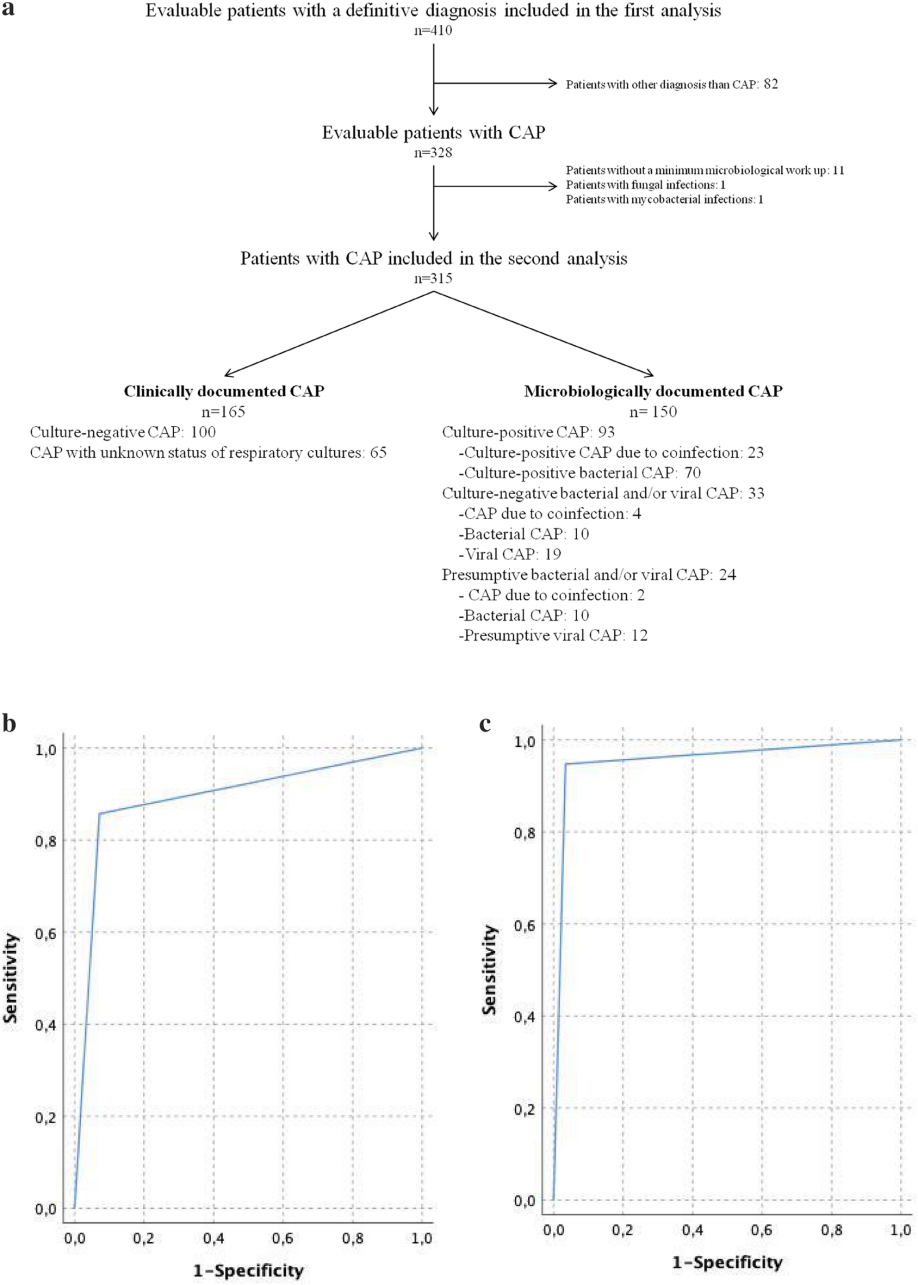
(**a**) Flow chart of patient enrollment and determination of the etiologies of community-acquired pneumonia. *CAP* community-acquired pneumonia, *LUS* lung ultrasound. Please see Supplement for discursive definitions of each cohort. (**b**) Receiver operating characteristic curve for predicting CAP caused by bacteria (all bacterial CAP vs viral CAP) by lung ultrasound. The area under the receiver operating characteristic curve = 0.89 (95% CI, 0.82%-0.97%). (**c**) Receiver operating characteristic curve for predicting bacterial coinfection among patients with CAP caused by viruses (CAP due to coinfection vs viral CAP) by lung ultrasound. Area under the receiver operating characteristic curve = 0.95 (95% CI, 0.86–1%).

Out of the 328 patients with a definitive diagnosis of CAP, 13 patients were excluded from the second analysis because a minimum microbiological work-up was not performed (11), they were diagnosed with mycobacterial infections (1), or they were diagnosed with fungal infections (1). Thus, 315 patients with CAP were included in the second analysis (Fig. 2a) and were stratified according to the availability and the results of the bacterial and viral bundles (Figure S1). The clinical characteristics of the patients with a definitive diagnosis of CAP are shown in Table 1. A total of 93 (30%) patients with CAP had culture-positive CAP (blue rectangles in Figure S1), and 133 (42%) patients with CAP had all culture-negative CAP (green rectangles); the cultural status of CAP could not be determined in the remaining 89 (28%) patients. A microbiological diagnosis was made in 150 (48%) of the 315 CAP patients (Fig. 2a), with 90 (29%) having bacterial infection alone (i.e., bacterial CAP, yellow rectangles), and 60 (19%) showing evidence of viral infection (Table 1). Among patients with viral infection, 19 (32%) had viral infection alone (i.e., viral CAP, red rectangles), 29 (48%) had evidence of mixed viral-bacterial infection (i.e., CAP due to coinfection, purple rectangles), and 12 (20%) were diagnosed with presumptive viral CAP (dotted gray rectangles). Independent of the viral bundle results, bacteria were implicated in 119 (79%) of the microbiologically documented CAP cases (i.e., all bacterial CAP, orange rectangles). All pathogens identified at the end of the work-up are available in Table S2. The proportion of patients exhibiting different LUS results and positive patterns according to the outcomes of the second analysis are shown in Table 3. Ninety-three (100) patients with culture-positive CAP and 126 (95%) patients with culture-negative CAP showed positive LUS results (*p* = 0.043). Pattern 1 was strongly associated with culture-positive CAP (*p* = 0.038). The same pattern was also more commonly found in patients with all bacterial CAP, CAP due to coinfection and bacterial CAP when they were individually compared with patients with viral CAP (all *p* < 0.001). The clinical characteristics of the patients with CAP who exhibited pattern 1, pattern 2, and negative LUS results are shown in Table S3. Pattern 1 was associated with the highest median body temperature, white blood cell count, and serum concentration of inflammatory biomarkers (C-reactive protein and procalcitonin). Compared to pattern 2, pattern 1 was significantly linked with clinical deterioration (*p* = 0.015) and mortality at 30 days (*p* = 0.006). All 8 patients with CAP who were showed false negative LUS results survived at 30 days; none of them were culture-positive at the end of the microbiological work-up. The performance of LUS in predicting all the outcomes of the second analysis is provided in Table 2. The receiver operating characteristic [ROC] curve and AUROC predicting all bacterial CAP and CAP due to coinfections by LUS are reported in Fig. 2b,c, respectively.

**Table 3:**
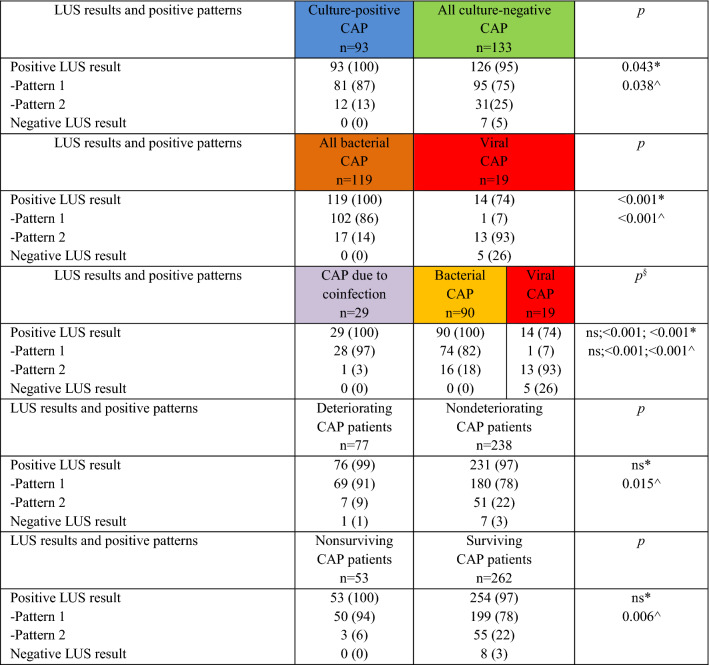
Lung ultrasound results and positive patterns according to the outcomes of the second analysis.

*LUS* lung ultrasound, *CAP* community-acquired pneumonia, *ns* not significant.

The color of the heading refers to the color assigned to the specific microbiological cohort of community-acquired pneumonia in Figure S1. Please see Supplement for discursive definitions of each cohort.

*Positive LUS result vs negative LUS result; ^pattern 1 vs pattern 2; ^§^*p* = X;X;X (CAP due to coinfection vs bacterial CAP, bacterial CAP vs viral CAP, CAP due to coinfection vs viral CAP).

## Discussion

This study assessed the usefulness of LUS performed at hospital admission in supporting physicians in several aspects of CAP management from diagnosis through the identification of patients in whom cultures are likely/unlikely to provide useful diagnostic information and of patients in whom empirical antibacterial therapy is mandatory to prognostication.

LUS was proven to be more useful than CXR in diagnosing CAP in patients with suspected LRTI in this work (Fig. 1b). Their AUROCs for predicting CAP were similar to those found in recent meta-analysis of clinical studies^3–6^. However, the diagnostic performance of LUS was worse than that of CCT (Fig. 1a): 9 patients with diagnoses other than CAP showed false positive results, and 8 patients with CAP showed false negative LUS results (Fig. 1a). These data are in line with those found in previous studies^3–6^. In our work, all the patients (9) with false positive LUS results received a diagnosis of lung cancer. Pattern 1 was identified in all of them. It is possible that some features of pattern 1 that are not specific to CAP may have misled the independent physicians who performed LUS, causing them to give misdiagnoses. For example, fluid bronchogram reflects not only airways filled with fluid but also secretions following airway obstruction. Several factors have been hypothesized to justify false negative LUS results, such as small-dimension echographic artifacts and their deep parenchymatous location (LUS may only detect consolidations reaching the pleura)^3–6^. We suspected that these features could be related to a specific cultural status and etiology of CAP. In fact, in the present work, out of the 8 patients with CAP not identified by LUS, 7 patients had culture-negative CAP (5 of them were proven to have viral CAP), and 1 patient was presumed to have viral CAP.

Although cultures could improve the quality of treatment in patients with CAP, guidelines do not support their routine collection in relation to the low quality of studies demonstrating clinical benefit^10^. Thus, the choice of whether to culture patients with CAP is challenging: it should be based on the severity of clinical presentation, local ecological considerations, and local antimicrobial stewardship processes^10^. We aimed to assess the usefulness of LUS in supporting this decision. In this study, among patients with CAP and known cultural status at the end of minimum microbiological work-up, a negative LUS result ruled out the need for cultures with a high negative predictive value (100%). This resulted in the identification at hospital admission of 3% of all the patients with CAP in whom cultures did not provide useful diagnostic information. Unfortunately, the predictivity of a positive LUS result for culture-positive status was poor in this study (PPV 42%).

Given the increasing occurrence of antibiotic resistance^10^, the possibility of predicting the etiology of CAP following hospital admission could represent an important step forward. In our work, the performance of LUS in predicting the need for empirical antibacterial therapy was excellent because pattern 1 ruled in CAP of bacterial etiology and bacterial coinfection with high PPVs (99% and 97%, respectively). Notably, in the latter cohort, pattern 2 exhibited a good performance for ruling out the need for antibacterial therapy (NPV: 93%). Host response seems to support the observed correlation of pattern 1 with CAP of bacterial etiology and that of pattern 2/negative LUS results with its viral etiology (Table S3). In fact, pattern 1 was associated with higher median serum concentrations of procalcitonin^13,14^ if it was individually compared with pattern 2 or negative LUS results (Table S3).

Determining whether a patient with CAP can be safely treated in an IMW or whether a higher acuity level of inpatient care is needed is an essential step in medical management. The severity of illness is one of the most critical factors in addressing this question. In this study, LUS was useful in identifying patients with CAP who may be safely managed in a low-intensity medical care setting. In fact, pattern 2 exhibited good performance in ruling out mortality due to CAP (NPV: 95%; Table 2). Additionally, none of the 8 patients with a definitive diagnosis of CAP exhibiting negative LUS results at IMW admission died, and only 1 of them experienced an increase in the number of dysfunctional organs during the 30 days of clinical follow-up. Even if the ability of pattern 1 to predict those adverse outcomes was poor (PPV 28% and 20%, respectively), pattern 1 was proven to be strongly linked with clinical deterioration (*p* = 0.015; Table 2) and mortality (*p* = 0.006). This result might also be explained by the fact that the vast majority (97%) of patients with CAP due to coinfection exhibited pattern 1 on LUS (Table S3). In fact, in recent studies, CAP with mixed bacterial and viral etiology has been associated with higher mortality than CAP caused solely by bacteria or viruses^20–23^. Whether LUS could augment existing criteria such as the well validated major and minor IDSA/ATS Severity Criteria^10^ should be addressed in future studies.

This work has several strengths. The use of LUS for diagnosis CAP was assessed in patients with suspected LRTI for the first time. Additionally, to enroll the patients we used clinical criteria which are standardised and reproducible^2^. In this study, the extension of microbiological work-up is clearly stated and broad. At least two sets of blood cultures drawn and about three other microbiological tests were performed for each patient with CAP to establish the etiology of infection (Table 1). The predictivity of LUS for culture-positive status and the bacterial etiology of CAP was assessed in 1) microbiological cohorts of patients in whom the definitive etiology of CAP could be ascertained with reasonable certainty and 2) in a clinical context in which the pretest probability of viral (19%) and mixed bacterial and viral infections (9%) were in line with those found in recent meta-analyses of epidemiological studies^23,24^.

Our study has limitations. First, CAP of different etiologies cannot be perfectly distinguished. For example, the identification of viral pathogens, particularly via nasopharyngeal swabs, does not necessarily indicate that viruses are the cause of CAP (it could be due to upper airway infection or asymptomatic shedding)^1^. All the studies in this field share the same limitation. Second, the number of patients with viral CAP was small. Thus, our results should be confirmed in large multicentric prospective studies. Third, we did not record the number of complicated pneumonia or those requiring surgical procedures^25^. Fourth, the choice to not detail the number, location, shape and size of the echographic artifacts may represent an oversimplification^26^. However, it could mitigate both the impact of different background expertise and of interobserver variability on LUS performance. In this respect, 50 recorded cine-loops that resulted positive for CAP in this study were rated (pattern 1 vs pattern 2) by two internal medicine residents with less than 6 months of experience with point-of-care ultrasonography: a substantial agreement was observed between the two observers (K = 0.75, 95%CI 0.55–0.96). Additionally, our condensed classification of all the positive LUS results may reduce the time need to perform LUS. This aspect is massively important in patients with bacterial CAP complicated by organ dysfunction in whom there is the need to start antibacterial therapy as quick as possible^27^. In this study, they represent the vast majority of the patients with CAP (Table S3). Finally, this fast categorization could prove crucial for the feasibility of LUS to predict the microbiological outcomes and to identify the most appropriate trajectories of CAP in future studies conducted in emergency setting.

## Conclusions

In this study, LUS performed at IMW admission was proven to be useful for ruling in the diagnosis and bacterial etiology of CAP and for ruling out mortality in patients with CAP. Larger prospective studies, performed in other settings, are warranted to support our findings.

## Supplementary Information

Supplementary Information.

## Data Availability

The datasets used and/or analyzed during the current study are available from the corresponding author on reasonable request.
