# Supplementary material for: Lung ultrasound may support internal medicine physicians in predicting the diagnosis, bacterial etiology and favorable outcome of community-acquired pneumonia
Source: Sci Rep. 2021 Aug 23;11:17016. doi: 10.1038/s41598-021-96380-x (PMC8382746; doi:10.1038/s41598-021-96380-x)
Supplement: Supplementary file 1 — Supplementary Information. [file 41598_2021_96380_MOESM1_ESM.docx]

**SUPPLEMENTARY INFO**

Supplementary information on inclusion/exclusion criteria of the first and second analysis, sample size, scanning techniques, definition of minimum microbiological work up, interpretation of microbiological results, microbiological definitions, definition of severity of CAP at hospital admission, definition of clinical deterioration within 30 days of IMW admission, characteristics of patients with suspected LRTI, typology and number of pathogens identified in CAP, clinical characteristics of the patients with CAP exhibiting pattern1, pattern 2 and negative LUS results, categorization of the patients with CAP according to the availability and results of the tests pertaining to bacterial and viral bundles.

**INCLUSION AND EXCLUSION CRITERIA**

FIRST ANALYSIS

INCLUSION CRITERIA

Adult patients (>18 years) with suspected lower respiratory tract infection (LRTI) at internal medicine ward (IMW) admission. Suspected LRTI was defined by cough with at least one of sputum production, dyspnea, wheeze or chest discomfort/pain and no alternative explanation (i.e., sinusitis and asthma). This acute illness had to be present for 21 days or less.

EXCLUSION CRITERIA

1) Patients with suspected LRTI who refused to participate in the study

2) Patients with suspected LRTI and suspected or witnessed aspiration

3) Patients with suspected LRTI who did not undergo either chest X-ray or lung ultrasound at IMW admission.

SECOND ANALYSIS

INCLUSION CRITERIA

Adult patients (>18 years) who received a definitive diagnosis of community-acquired pneumonia (CAP) by the physician in charge at the end of clinical work-up.

EXCLUSION CRITERIA

1) Patients with CAP without a minimum microbiological work-up

2) Patients with CAP with a minimum microbiological work-up who were diagnosed with an infection caused by pathogens other than bacteria and viruses.

**SAMPLE SIZE**

We calculated the minimum sample size necessary to identify a difference between two AUC ROC curves derived on the same subjects; the input parameters for the sample size calculation were AUROC_1_=0.80, AUROC_2_=0.70, a power of 80% and a significance level of 5% and a ratio of sample sizes in negative/positive groups of 0.3.  With these parameters, 236 cases are required in the positive group and 71 in the negative group, giving a total of 307 cases.

**SCANNING TECHNIQUES**

All ultrasounds were performed at the bedside. Anterolateral and posterior scans of the thorax were performed with both convex and linear probe. LUS was performed on the whole chest, just laying the probe in the intercostal spaces, avoiding the ribs. The probe was positioned both longitudinally, perpendicular to the ribs, and obliquely, along the intercostal spaces. The posterior areas were studied in the lateral decubitus or in the sitting position according to clinical status. Each hemithorax was divided into five areas: two anterior, two lateral, one posterior, for a total of 10 areas. The anterior chest wall was marked off from the parasternal line to the anterior axillary line. This zone was split into an upper region (from the collar bone to the second-third intercostal space) and a lower region (from the third intercostal space to the diaphragm). The lateral area (anterior to posterior axillary line) was split into upper and lower halves. Finally, the posterior area was identified from the posterior axillary line to the paravertebral line.

**DEFINITION OF MINIMUM MICROBIOLOGICAL WORK-UP**

Minimum microbiological work-up was considered completion of bacterial and viral bundles:

Bacterial bundle

The bacterial bundle was considered completed when at least two sets of blood cultures were drawn (<24 h from IMW admission) and

- at least one respiratory sample was drawn (<24 h from IMW admission) for detection of cultures and/or
- at least one urine sample was taken for detection of *Streptococcus pneumoniae* and *Legionella pneumophila* (antigens) and/or
- at least one serum sample was taken for detection of *Mycoplasma pneumoniae* and *Chlamydophila pneumoniae* (serologies)

Viral bundle

The viral bundle was considered completed when

- at least one nasopharyngeal swab was collected for detection of influenza, parainfluenza viruses, respiratory syncytial virus, adenovirus (polymerase chain reaction) and/or
- at least one serum sample was collected for detection of the same respiratory viruses (serologies)

**INTERPRETATION OF MICROBIOLOGICAL RESULTS**

Adequate sputum sample

- <10 epithelial cells and
- ≥ 25 white blood cells per low power field.

Positive cultures

- A microorganism was cultured from blood (in this study, a single blood culture positive for organisms consistent with skin flora [coagulase-negative Staphylococci, Corynebacterium spp, and alpha-hemolytic Streptococci] was considered contaminated) and/or
- a microorganism was cultured from pleural fluid and/or
- sputum yielded growth of ≥ 10^6^ colony forming units per milliliter and/or
- bronchoalveolar lavage yielded growth of ≥ 10^5^ colony forming units per milliliter.

Serologies for atypical bacteria and respiratory viruses

Diagnosis of *Mycoplasma pneumonia*, *Chlamydophila pneumoniae*, influenza, parainfluenza viruses, respiratory syncytial virus, and adenovirus infection was carried out by pairing sera on admission and 3-10 weeks late. High titers of IgM in the acute phase were also accepted for the diagnosis of *Mycoplasma pneumoniae* (any positive titer) and *Chlamydophila pneumoniae* (≥ 1:32)

**MICROBIOLOGICAL DEFINITIONS**

Microbiologically documented CAP: CAP with positive microbiological results independent of cultural status. This cohort was further stratified into the following groups

- Culture-positive CAP: patients with CAP in which respiratory samples and/or blood cultures turned positive. This cohort included not only patients with CAP in which bacteria were proven to be the sole etiology of CAP by cultures but also patients with CAP in whom bacteria were identified by cultures and viruses were simultaneously identified by diagnostic tests complementary to cultures (i.e., serologies, PCR on nasopharyngeal swabs, and urinary antigens). The latter groups were named culture-positive bacterial CAP and culture-positive CAP due to coinfection, respectively.
- Culture-negative bacterial and/or viral CAP: patients with CAP from whom
  - respiratory cultures were drawn, resulted adequate and turned negative and
  - blood cultures were drawn and turned negative

In this group, the etiology of CAP was documented by diagnostic tests complementary to cultures (i.e., serologies, PCR on nasopharyngeal swabs, and urinary antigens)

- Presumptive bacterial and/or viral CAP: patients with CAP from whom
  - respiratory samples were not drawn or were inadequate and
  - blood cultures turned negative

In this cohort, the etiology of CAP was documented by diagnostic tests complementary to cultures.

Clinically documented CAP: patients with CAP without any microbiological documentation. This cohort was split in

- Culture-negative CAP: patients with CAP for whom
  - respiratory cultures were drawn, were adequate and turned negative and
  - blood cultures turned negative and
  - the diagnostic test complementary to cultures turned negative.
- CAP with unknown status of respiratory cultures: patients with CAP for whom
  - respiratory samples were not drawn or were inadequate and
  - blood cultures turned negative and
  - the complementary test to cultures turned negative.

Main microbiological cohorts

All bacterial CAP: patients with CAP in whom a bacterial infection was documented (regardless of the method of identification) independently from the results of the viral bundle.

CAP due to coinfection: patients with CAP for whom a mixed bacterial (regardless of the method of identification) and viral infection was documented.

Viral CAP: patients for whom CAP a viral infection was documented and cultures on high-quality respiratory samples and cultures on blood turned negative.

All culture-negative CAP: patients with CAP for whom both cultures on high-quality respiratory samples and cultures on blood turned negative independently from the results of the viral work-up.

**SEVERITY OF CAP AT HOSPITAL ADMISSION**

At hospital admission, the number of organ dysfunctions was measured. Organ dysfunction was considered present when any of the following criteria were met:

- SaO2< 90%* or PaO_2_/FiO_2_ <300* or acute respiratory distress syndrome (respiratory dysfunction),
- increase in serum creatinine of 0.3 mg/dl or ≥ 1.5-fold from baseline within 48 h (renal dysfunction),
- platelet count < 100000/mm^3^ ** (hematologic dysfunction),
- apTT >60 sec** or INR> 1.2** or disseminated intravascular coagulation (hemostasis dysfunction),
- total bilirubin > 4 mg/dl* or INR > 1.5** (liver dysfunction),
- any form of mental change, inattention, and disorganized thinking indicative of encephalopathy or delirium or any degree of Glasgow Coma Scale worsening (neurologic dysfunction), and
- cardiac arrhythmias*** as high-rate atrial fibrillation, high-rate narrow complex, ventricular tachycardia, acute coronary syndromes, and acute decompensated heart failure*** (cardiovascular dysfunction). High-rate atrial fibrillation or narrow complex tachycardia were observed in patients who were clinically or hemodynamically unstable (i.e., myocardial ischemia, pulmonary edema, or hypotension).

^*^not known to be chronic

^**^not known to be chronic or due to medications

^***^ during the concurrent hospital stay for sepsis but not primary diagnosis

**CLINICAL DETERIORATION WITHIN 30 DAYS OF HOSPITAL ADMISSION**

Clinical deterioration within 30 days of hospital admission was defined as any increase in the basal number of organ dysfunctions.

Table S1. Patients with suspected lower respiratory tract infection: characteristics at internal medicine ward admission, and definitive diagnosis made by physician in charge at the end of clinical work-up.

| Characteristics | n=  410 (100) |
| --- | --- |
| **Demographics**  Male  Median age (IQR) | 213 (52)  81 (75-87) |
| **Comorbidities**  Diabetes  Chronic heart failure  Previous acute myocardial infarction  Cancer  -Solid  -Haematologic  Chronic liver disease  Chronic pulmonary disease  Chronic kidney disease  Dementia  Chronic rheumatologic disease  AIDS | 95 (23)  105 (26)  62 (15)  45 (11)  22 (5)  5 (1)  195 (48)  79 (19)  71 (17)  12 (3)  1 (0) |
| **Severity at IMW admission**  Median number of organ dysfunctions | 1 (1-2) |
| **Radiological work-up**  Chest X-ray  Lung ultrasound  Computed chest tomography | 410 (100)  410 (100)  58 (14) |
| **Microbiological work-up**  At least two sets of blood cultures  At least one:  -respiratory sample for cultures  - urine sample for pneumococcal and *Legionella* antigens  -serum sample for atypical bacteria and respiratory viruses (serologies)  -nasopharyngeal swab for respiratory viruses (PCR) | 410 (100)  258 (63)  369 (90)  330 (80)  265 (64) |
| **Defintive diagnosis at the end of clinical work-up**  Lower respiratory tract infections  -Pneumonia  -Other lower respiratory tract infections  -Acute exacerbation of COPD  -Acute exacerbation of bronchiectasis  -Acute bronchitis  Other diagnosis | 406 (99)  328 (80)  78 (19)  66 (85)  3 (4)  9 (11)  4 (1) |

List of abbreviations: IQR=interquartile range, AIDS= acquired immune deficiency syndrome, IMW= internal medicine ward, PCR=polymerase chain reaction, COPD =chronic obstructive pulmonary disease.

Table S2. Typology and number of pathogens identified in patients with community-acquired pneumonia.

| Etiology of microbiologically documented CAP | n |
| --- | --- |
| **BACTERIA** | 142 |
| Typical bacteria | 127 |
| -Gram negative | 70 |
| -Pseudomonas spp | 15 |
| -Klebsiella spp | 14 |
| -*Haemophilus influenza* | 8 |
| -*Escherichia coli* | 8 |
| -Enterobacter spp | 8 |
| -*Acinetobacter baumanii* | 5 |
| -*Morganella morganii* | 4 |
| -*Serratia marcescens* | 4 |
| -Citrobacter spp | 2 |
| -Other gram negative | 2 |
| -Gram positive | 57 |
| -Staphylococcus spp  -*Staphylococcus aureus*  -coagulase-negative Staphylococci | 39  38  1 |
| -Streptococcus spp  -*Streptococcus pneumoniae*  -Other Streptococcus species | 14  9  5 |
| -Other gram positive bacteria | 4 |
| Atypical bacteria | 15 |
| -*Chlamydophila pneumoniae* | 10 |
| -*Mycoplasma pneumoniae* | 5 |
| **VIRUS** | 53 |
| -Influenza | 32 |
| -Respiratory syncytial virus | 16 |
| -Parainfluenza | 5 |
| -Herpesvirus | 5 |
| Total | 195 |

List of abbreviations: CAP=community-acquired pneumonia.

Table S3. Clinical characteristics of the patients with community-acquired pneumonia exhibiting pattern 1, pattern 2, and negative LUS results.

| Characteristics | Pattern 1  n=249 (79) | Pattern 2  n=58 (19) | LUS negative results  n=8 (2) | *p* |
| --- | --- | --- | --- | --- |
| **Demographics**  Male  Median age (IQR) | 133 (43)  83 (77-88) | 29 (9)  80 (73-84) | 5 (1)  87 (81-90) | ns;ns;ns  ns;ns;ns |
| **Comorbidities**  Diabetes  Chronic heart failure  Previous acute myocardial infarction  Cancer  -Solid  -Haematologic  Chronic liver disease  Chronic pulmonary disease  Chronic kidney disease  Dementia  Chronic rheumatologic disease | 57 (23)  68 (27)  41 (16)  34 (14)  16 (6)  2 (1)  92 (37)  54 (22)  53 (21)  6 (2) | 13 (22)  16 (28)  8 (14)  6 (10)  4 (7)  1 (2)  30 (5)  6 (10)  6 (10)  2 (3) | 4 (50)  3 (37)  2 (25)  0 (0)  0 (0)  0 (0)  3 (37)  1 (12)  2 (25)  0 (0) | ns;ns;ns  ns;ns;ns  ns;ns;ns  ns;ns;ns  ns;ns;ns  ns;ns;ns  ns;ns;ns  ns;ns;ns  ns;ns;ns  ns;ns;ns |
| Median body temperature (°C) | 37.4 (36.6-38) | 37 (37-37.8) | 37.2 (36.5-38) | 0.023;ns;ns |
| **Laboratory at IMW ward admission**  Median white blood cell count/mm^3^  Median C-reactive protein (mg/dl) (IQR)  Median procalcitonin (ng/ml) (IQR) | 11.18 (8.26-15.20)  84.55 (34.8-164.27)  0.38 (0.12-2.02) | 10.13 (7.86-12.57)  43.1 (12.7-97.3)  0.07 (0.04-0.21) | 8.98 (6.33-11.21)  29.95 (5.65-59.42)  0.05 (0.04-0.19) | ns;ns;ns  0.001;ns;0.015  <0.001;ns;0.014 |
| **Severity at IMW admission**  Median number of organ dysfunctions | 1 (1-2) | 1 (1-2) | 2 (1-3) | 0.003;ns;ns |
| **Cultural status**  Culture-positive CAP  All culture-negative CAP  **Etiology**  All bacterial CAP  -CAP due to coinfection  -Bacterial CAP  Viral CAP | 81 (87)  95 (72)  102 (86)  28 (97)  74 (82)  1 (5) | 12 (13)  31 (23)  17 (14)  1 (3)  16 (18)  13 (69) | 0 (0)  7 (5)  0 (0)  0 (0)  0 (0)  5 (26) | ns;ns;0.043*  <0.001;<0.001;<0.001^  <0.001;ns;<0.001^§^ |
| **Outcome within 30 days of IMW admission**  Worsening of clinical conditions  Mortality | 69 (28)  50 (16) | 7 (12)  3 (1) | 1 (12)  0 (0) | 0.032;ns;ns  0.02;ns;ns |

List of abbreviations: IQR=interquartile range, IMW= internal medicine ward, ns=not significant.

Figure legend: (x;x;x)=pattern 1 vs pattern 2, pattern 2 vs LUS negative results, pattern 1 vs LUS negative results.*=culture-positive CAP vs all culture-negative CAP; ^=all bacterial CAP vs viral CAP; ^§^=CAP due to coinfection vs viral CAP.

Figure S1. Categorization of the patients with community-acquired pneumonia according to the availability and results of the tests pertaining to bacterial and viral bundles.


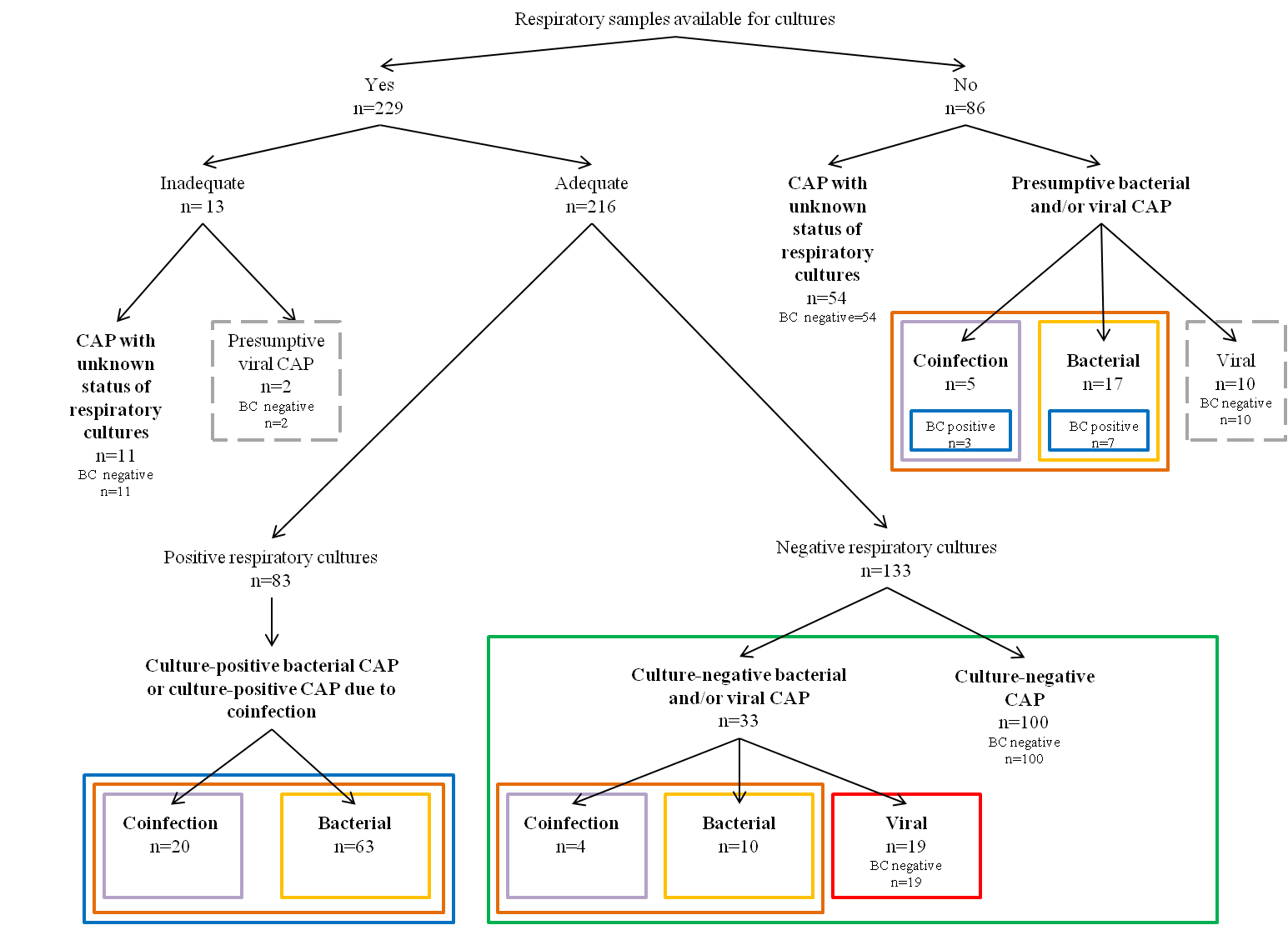


List of abbreviations: CAP=community-acquired pneumonia.

Figure legend: CAP due to coinfection (purple rectangles), bacterial CAP (yellow rectangles), culture-positive CAP (blue rectangles), all bacterial CAP (orange rectangles), viral CAP (red rectangle), presumptive viral CAP (dotted gray rectangles), and all culture-negative CAP (green rectangle).

Please see Supplement for discursive definitions of each cohort.
